# (-)-Epicatechin Promotes Epigenetic and Metabolic Changes in an Obesity Model

**DOI:** 10.3390/biom16030343

**Published:** 2026-02-24

**Authors:** Javier Pérez-Durán, Miguel Ortiz-Flores, Sarai Mendoza-Bustos, Yuridia Martínez-Meza, Aglae Luna-Flores, Guillermo Ceballos, Nayelli Nájera

**Affiliations:** 1 Reproductive and Perinatal Health Research Department, Instituto Nacional de Perinatología Isidro Espinosa de los Reyes, Mexico City 11000, Mexico; djavier40@gmail.com; 2 Escuela Superior de Medicina, Instituto Politécnico Nacional, Mexico City 11340, Mexico

**Keywords:** DNA methyltransferases, obesity, (-)-epicatechin, DNA methylation

## Abstract

Background: Obesity is a multifactorial chronic disease resulting from sustained energy imbalance and modulated by environmental and demographic factors, and it is associated with numerous comorbidities. DNA methylation is an epigenetic modification associated with obesity. Modulation of DNA methylation is a viable target for obesity control strategies. The flavanol (-)-epicatechin (EC) exerts beneficial effects in overweight individuals, suggesting that EC may influence gene regulation through signaling pathways and epigenetic mechanisms. We evaluated whether EC modulates obesity-associated DNA methylation changes using complementary in silico, in vitro, and in vivo approaches. Methods. In silico analyses were performed to explore potential EC interactions with the DNA methyltransferases DNMT1, DNMT3A, and DNMT3B. DNMT activity was measured in nuclear extracts of 4T1 cells in the presence of EC. Finally, in a C57BL/6 mouse model of diet- induced obesity, we assessed global DNA methylation and the expression of the DNA methyltransferases, as well as metabolism-related genes; peroxisome proliferator-activated receptor gamma coactivator 1 alpha (*Pgc-1α*), pyruvate dehydrogenase kinase isozyme 4 (*Pdk4*), and nuclear factor erythroid 2–related factor 2 (*Nrf2*) and relative mitochondrial DNA content (mtDNA/nDNA ratio) in visceral adipose tissue (VAT) and skeletal muscle. Results. EC showed stable in silico interactions within catalytic/cofactor-binding regions of DNMTs and inhibited DNMT activity in vitro in a concentration-dependent manner. In vivo, the obesogenic diet reduced global DNA methylation and decreased transcript levels of *Dnmt1*, *Dnmt3a*, and *Dnmt3b* in skeletal muscle and adipose tissue. EC counteracted obesity-associated DNA methylation changes in skeletal muscle, restoring global methylation and Dnmt expression toward control levels, whereas effects in VAT were limited. EC increased mitochondrial DNA content. Discussion. In silico and enzymatic data suggest that EC may bind DNMT active sites and inhibit DNMT activity in a concentration-dependent manner, supporting a role for EC in obesity-related epigenetic remodeling, particularly in skeletal muscle. EC also increased relative mitochondrial DNA content in VAT and skeletal muscle despite no obesogenic diet effect on relative mitochondrial abundance, consistent with favorable mitochondrial modulation. In conclusion, EC is an epigenetic modulator and may have positive effects in obesity related dysfunctional tissues.

## 1. Introduction

Obesity is a multifactorial disease characterized by an excessive accumulation of body fat that contributes to the development of many associated comorbidities, such as type 2 diabetes (T2D), cardiovascular diseases, dyslipidemia, hypertension, liver steatosis, or metabolic syndrome [1,2,3,4]. Obesity and associated metabolic imbalances result from complex interactions between unhealthy environments, such as dietary and sedentary lifestyles, and demographics (age and sex). Epigenetic regulation may be a molecular mechanism linking these risk factors [5].

DNA methylation is an epigenetic modification that has consistently been linked to a variety of human diseases. This methylation is a dynamic process that is potentially reversible, reflects environmental exposures, can predict disease onset, and is a determinant of chromosomal inactivation [6].

DNA methylation occurs by adding a methyl (CH3) group to a cytosine. This reaction is catalyzed by a family of enzymes called DNA methyltransferases (DNMT1, DNMT3a, and DNMT3b). Two main mechanisms through which methylated cytosines (5mCs) exert their potential to modify gene activity have been proposed: (1) physically block the binding of transcription factors, resulting in gene silencing, or (2) attract methyl-binding proteins that can recognize 5mCs and cause changes in gene expression [7].

Beyond changes in lifestyle (increased mobility) and dietary habits that tend to reduce caloric imbalance, controlling obesity remains a major challenge, and the search for molecules capable of modulating DNA methylation safely and without secondary effects is a growing field. In this regard, we and others have been showing, in animals and humans, that the flavanol epicatechin (EC) or epicatechin-enriched products induce positive effects in overweight subjects [8] or cause the browning of fat in animal models of obesity or human adipocytes [9], suggesting that EC affects gene regulation through mechanisms that involve receptor activation, epigenetic changes, and more.

EC is part of a family of secondary metabolites known as flavonoids with a 15-carbon structure (two phenyl rings and a heterocyclic ring; C6-C3-C6) found in various vegetal products, such as fruits and legumes. EC has been associated with improvements in markers of inflammation, vascular function, and muscle structure and function [10].

Up to this point, the mechanisms described for EC are associated with the activation of intermediates of specific signaling pathways, such as the Mitogen-activated protein kinases (MAPKs), protein kinase B (Akt), and 5′-adenosine monophosphate-activated protein kinase (AMPK) [11], which depend on transmembrane receptor activation. We have shown that EC selectively binds to the Apelin receptor, inducing many of its effects through its activation [12].

We propose that EC can modulate obesity-associated epigenetic modifications. In this work, we analyze this possibility using several approaches: (1) in silico analysis exploring the possible interaction of EC with DNA methyltransferases DNMT1, DNMT3a, and DNMT3b; (2) exploring in vitro the EC effects on DNMT activity; (3) analyzing the EC effects in a mice model of obesity exploring DNA methylation levels and *Dnmt1*, *Dnmt3a*, and *Dnmt3b* expression levels; (4) analyzing EC-induced changes in the expression of peroxisome proliferator-activated receptor gamma coactivator 1 alpha (*Pgc-1α*), pyruvate dehydrogenase kinase isozyme 4 (*Pdk4*) and nuclear factor erythroid 2–related factor 2 (*Nrf2*); and (5) analyzing changes in relative mtDNA content (mtDNA/nDNA ratios).

## 2. Materials and Methods

The in silico analysis was performed following established methodologies, briefly [13,14,15]:

### 2.1. Molecular Docking

The 3D structure of mouse DNA methyltransferases 3a and 3b (DNMT3a and 3b) was modeled by homology with Swiss-Model software [15], using its human isoforms as templates. For the analysis of DNMT1, the crystal structure of mouse DNA methyltransferase-1 complexed with AdoHcy (SAH) was used (PDB ID: 3AV5). A first docking approach was performed using Smina software with a local script that determines the ligand-protein complex’s most frequent and best conformation after 1000 independent assays, centered on SAH coordinates, a well-known inhibitor, with a search space of 20 × 20 × 20 Å. The EC structure was obtained from PubChem, and its conformation was optimized using the Merck Molecular Force Field (MMFF94) in OpenBabel [14], as for the SAH structure used for docking validation. Finally, the best ligand conformation was determined based on docking scores and ligand interactions, and these coordinates were used as the initial configuration for the molecular dynamics simulation.

### 2.2. Molecular Dynamics

The best ligand-receptor conformation was subjected to 100 ns of molecular dynamics (MD) simulation in GROMACS 2021.4, using the CHARMM36-Jul2020 force field [13]. The ligand–protein conformation was assembled within a dodecahedron with a minimum distance of 1 nm from the protein edge and with periodic boundary conditions. After solvation using the simple point charge (SPC) and transferable intermolecular potential with 3 points (TIP3P) water models, the solvent molecules were replaced with 0.15 M NaCl and neutralized with counterions. The system was minimized using the steepest-descent method with 50,000 steps and a maximum force of 10 kJ/mol. The system was equilibrated in the canonical ensemble (NVT) for 100 ps, followed by isothermal-isobaric ensemble (NPT) equilibration for 100 ps with the protein, during which the ligand position was restrained. 100 ns of the simulation were carried out in the NPT ensemble at 300 K and 1 atm, employing the V-rescale method for temperature coupling and the Parrinello-Rahman method for pressure coupling [16]. The Particle Mesh Ewald method [17] was used to compute long-range electrostatic interactions with a non-bonded cutoff of 10 Å, and the linear constraint solver (LINCS) algorithm was employed, with interactions restricted to 10 Å using the Verlet cutoff scheme. The leap-frog algorithm was used to compute the equation of motion with a time step of 2 fs.

After completing molecular dynamics simulations for each protein, the time at which hydrogen bonds formed between EC and the catalytic and cofactor-binding residues of the evaluated isoforms was calculated.

### 2.3. DNMTs Activity Assay

Experiments were designed to analyze the effects of EC on isolated DNMT. Briefly:

Cultured cells with reported enzyme activity were used as the enzyme source. Specifically, 4T1 cells, a breast cancer cell line derived from a BALB/c mouse mammary tumor (ATCC CRL-2539), were employed. Cells were cultured in 30 cm Petri dishes at 37 °C and 5% CO_2_, supported in RPMI-1640 medium supplemented with 2% fetal bovine serum (FBS) and 1% antibiotics. Cells were scraped from culture plates, and the nuclear protein was subsequently extracted using the Nuclear Extraction Kit (ab113474, Abcam, Waltham, MA, USA). 7.5 mg of nuclear protein was used to measure the activity of DNMTs in the absence and presence of EC [1, 1.5, 2, 2.5, 5, 10, and 15 µM]. The enzymatic activity was determined using the DNMT activity Quantification kit (ab113467, Abcam, Waltham, MA, USA) according to the manufacturer’s instructions, with absorbance measured at 450 nm. The tests were carried out in triplicate.

### 2.4. Murine Model of Obesity

#### Ethical Approval

This study was approved by the institutional committee (INPER # 2018-1-160) and followed the guidelines of Mexican Official Standard NOM-062-ZOO-1999; Institutional research and ethics committees approved the protocol**:** Procedures and technical specifications for the production and animal care complied with the recommendations of Guide for the Care and Use of Laboratory Animals of the National Institutes of Health (Institute of Laboratory Animal Resource (US). Committee on Care and Use of Laboratory Animal 2011).

16 C57BL/6 mice were randomly assigned to 2 groups: (1) Control: 4 mice were fed with standard chow (LabDiet’s Rodent 5001) and water ad libitum; (2) Experimental: 12 mice were fed with a high-fat/obesogenic diet (Harlan Co., Indianapolis, IN, USA) as reported in [18]; body weight was measured every week; obesity was defined as >20% of body weight growth compared to the animals fed with standard chow. On day 42, the obese mice were randomly assigned to two groups: 6 mice continued the obesogenic diet and received vehicle by gavage for 20 days, and 6 mice received the obesogenic diet and epicatechin (1 mg/kg/day) by gavage for 20 days. Mice were maintained at room temperature and on a 12 h light/dark cycle. Perivisceral fat and skeletal muscle (gastrocnemius) were extracted and placed in Trizol and immediately processed.

### 2.5. Expression Assays

Total RNA was extracted from visceral fat (VAT) and skeletal muscle according to the supplier’s instructions using the Direct-zol^TM^ Total RNA kit from Zymo Research^®^ (Irvine, CA, USA). The concentration and purity were subsequently quantified in a NanoDrop 2000 (Thermo Fisher Scientific, Waltham, MA, USA). cDNA was synthesized using the qPCRBIO cDNA Synthesis Kit (PCR Biosystems Inc., East Hartford, CT, USA). The relative expression of the selected genes was determined using the SYBR Green reagent (BIO-SyGreen; Bio-Rad, Hercules, CA, USA) on a QuantStudio Real-Time PCR Detection System (Thermo Fisher, Waltham, MA, USA). The oligonucleotides used are shown in Table 1.

The PCR conditions were 95 °C for 20 s, followed by 40 cycles of 15 s at 95 °C and 30 s at 60 °C. Assays were performed in triplicate, and Actb mRNA was used as a constitutive gene for amplification. The comparative CT method (∆∆CT) normalized the results to the control.

### 2.6. Statistical Analysis

All results are presented as mean ± standard error of the mean (SEM) and analyzed by unpaired *t*-test to determine differences between each group’s means or by one-way ANOVA using multiple comparisons, followed by Tukey’s test to determine differences or Kruskal–Wallis test in case of nonparametric data; *p*-values < 0.05 were considered statistically significant. All analyses were performed in triplicate on the specified number of animals (*n* = 4 for the control group, *n* = 6 for OBS, and *n* = 6 for OBS + EC). IC_50_ (the concentration required to inhibit enzymatic activity by 50%) was determined by nonlinear regression using a 4-parameter logistic model, based on the evaluated concentration range (1–15 µM) and triplicate measurements.

## 3. Results

### 3.1. In Silico Analysis

**Figure 1:**
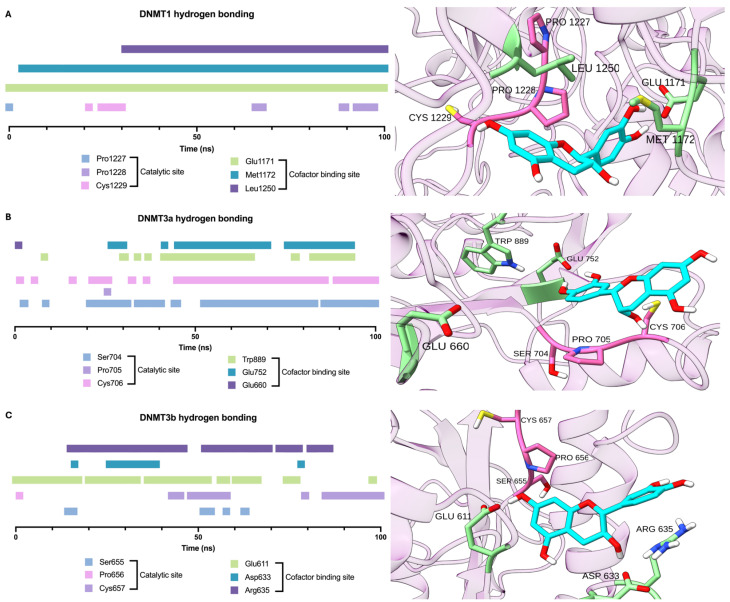
Analysis of the necessary time to form hydrogen bonds between EC and DNA methyltransferases (**A**) DNMT1, (**B**) DNMT3a, and (**C**) DNMT3b, with the most representative cluster for each conformation.

The results of the in silico analysis (Figure 1) showed similar EC and SAH binding scores for DNMT1, DNMT3a, and DNMT3b, suggesting that EC may act as an antagonist. This assumption was reinforced by the observation that EC binds to Pro705 and Cys706 in DNMT3a and to Pro656 and Cys657 in DNMT3b, which are in the enzymes’ catalytic sites.

### 3.2. DNMT Activity

**Figure 2:**
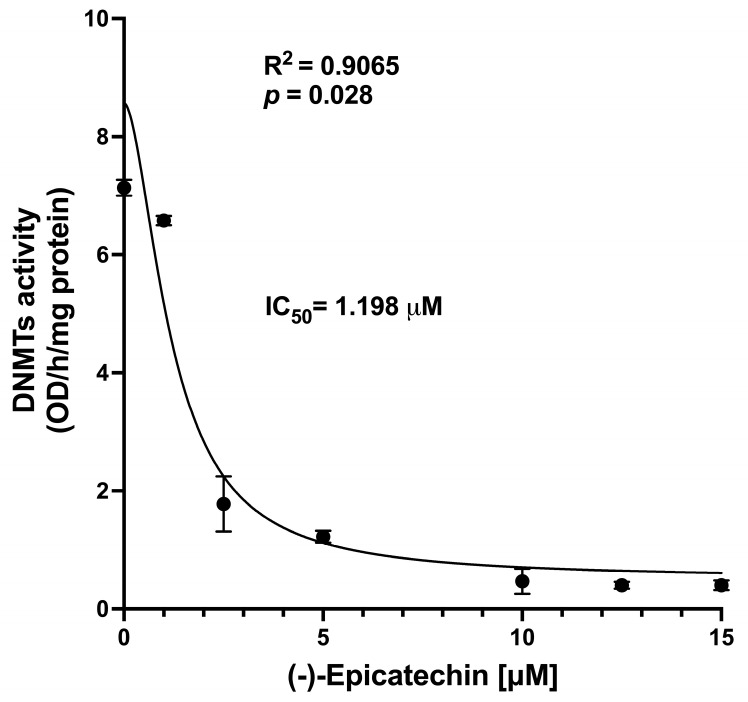
Analysis of EC-induced, concentration-dependent inhibition of DNMT enzymatic activity in 4T1 cells’ nuclear extracts. Data are represented as means ± sem. Results represent assays performed in triplicate.

The analysis was performed using nuclear extracts from 4T1 cells; this approach does not allow for discrimination among the three DNMT isoforms. The results show that EC induced a concentration-dependent change in DNMT activity measured in nuclear extracts (Figure 2). This result should be interpreted with caution, as it was not obtained using isolated enzymes.

The EC concentration causing 50% inhibition (IC_50_) of activity was 1.198 μM; these results are consistent with in silico results showing that EC may interact with DNMTs.

### 3.3. Animal Model of Obesity

After demonstrating (in silico and in vitro) that EC can inhibit methylation through DNMT inhibition, we investigated its effects in vivo in a murine obesity model, including the epigenetic changes induced by an obesogenic diet and any EC-induced effects that modulate these changes.

**Figure 3:**
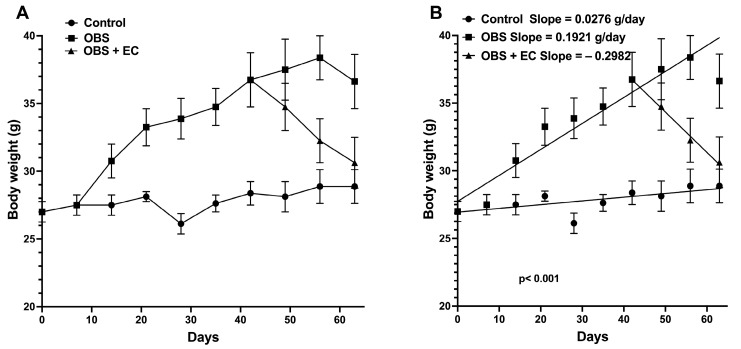
Analysis of body weight changes induced by HDF for 42 days, and the EC induced a decrease in body weight changes. (*n* = 4 for control group and *n*= 12 for HFD.) (**A**) Comparison of body weight changes. (**B**) Linear analysis of body weight changes. Data are represented as means ± sem.

First, we induced obesity in mice by feeding them a high-fat diet (HFD) for 6 weeks. Figure 3A,B illustrate the changes in body weight resulting from a high-fat diet (HFD) compared to a standard chow diet. Figure 3B shows that the positive slopes of body weight changes in the control group (standard chow), 0.0276 g/day, are different (*p* < 0.0001) from the slope of the HFD group, 0.1921 g/day, indicating that animals are larger and essentially obese compared to the control group. Interestingly, EC [1 mg/kg/day] for 3 weeks significantly decreases body weight, with a negative slope of −0.2982 (Figure 3).

### 3.4. DNA Methylation in Mouse Tissues

Since obesity induces deleterious (dysfunctional) changes in skeletal muscle (including an obesity-related sarcopenia) and in visceral fat (essentially white fat), causing hypoxia, meta-inflammation, and altering the levels of Leptin (may be related to Leptin resistance) accompanied by a decrease in mitochondrial number and function, [19,20,21,22,23] we decided to explore the EC effects in the obese model.

At the end of the experimental setup, global DNA methylation (% 5mc) and expression changes in target genes were analyzed in perivisceral fat and skeletal muscle (gastrocnemius) to explore the obesogenic diet-induced changes and EC-induced effects.

**Figure 4:**
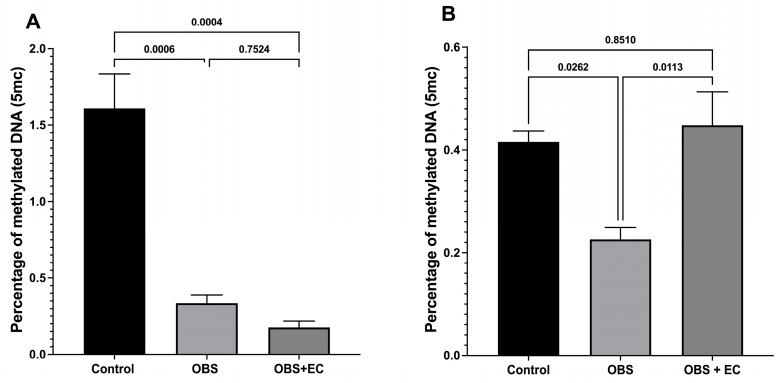
Analysis of DNA methylation percentage in control mice and induced changes by ingestion of HDF and the EC-induced modifications in DNA methylation in (**A**) visceral fat and (**B**) skeletal muscle (*n* = 4 for control group, *n* = 6 for OBS, and *n* = 6 for OBS + EC). Data are represented as means ± SEM.

Figure 4A shows the percentage of methylated DNA in VAT. Results showed a significant decrease in DNA methylation in the obesogenic and obesogenic diets plus EC groups. Figure 4B shows the DNA methylation in skeletal muscle. The methylation percentage is lower than in VAT in the control group. Notably, the obesogenic diet reduces DNA methylation, rather than increasing it as expected, and treatment with EC prevents this decrease, as DNA methylation does not differ from that of the control group. These results suggest specific and differential EC effects among mouse tissues.

### 3.5. Dnmts Expression

**Figure 5:**
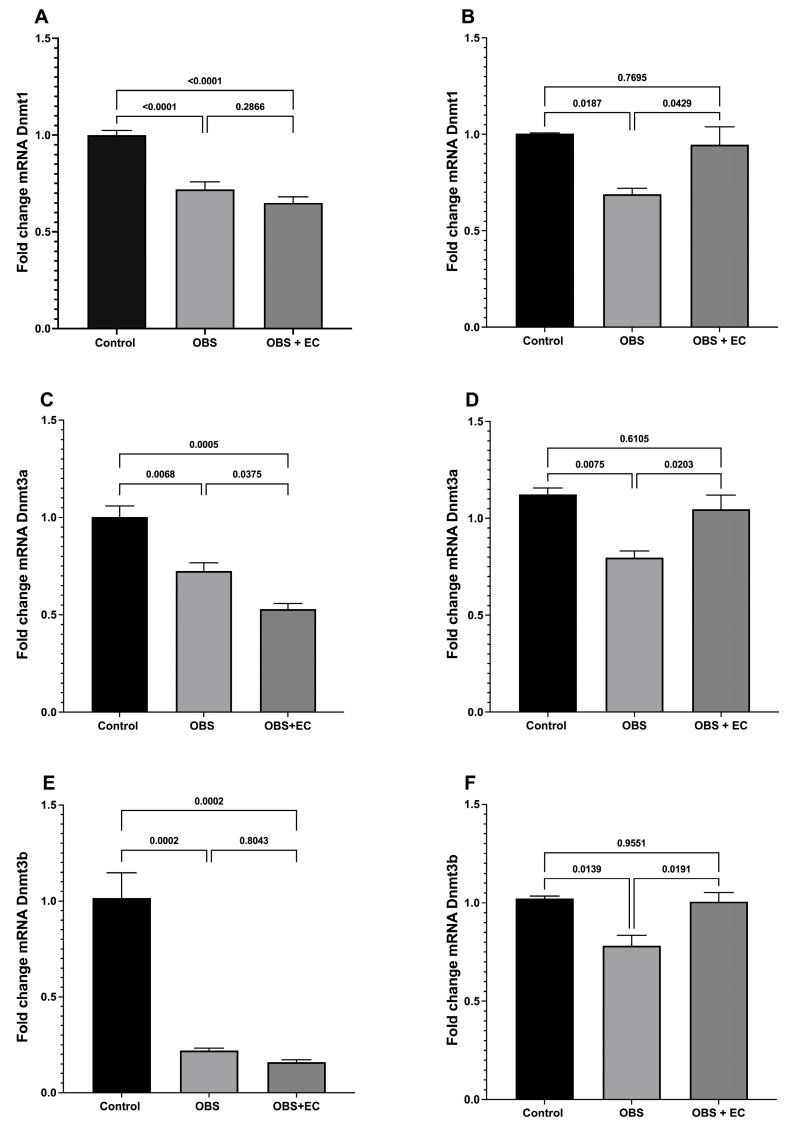
Analysis of *Dnmt1* expression (**A**,**B**); *Dnmt3a* expression (**C**,**D**), and *Dnmt3b* expression (**E**,**F**) in visceral fat (**A**,**C**,**E**) and skeletal muscle (**B**,**D**,**F**). Control = mice fed with standard chow; OBS = mice fed with HFD, OBS + EC = mice fed with HFD plus EC treatment (*n* = 4 for control group, *n* = 6 for OBS, and *n* = 6 for OBS + EC). Data are represented as means ± SEM.

Figure 5 shows fold changes in *Dnmt1*, *Dnmt3a*, and *Dnmt3b* mRNA levels in VAT and skeletal muscle. In visceral fat, *Dnmt1* mRNA levels (Figure 5A) were significantly lower in the obesogenic diet and obesogenic diet plus EC groups. A similar decrease was observed for *Dnmt3a* (Figure 5C) and *Dnmt3b* (Figure 5E).

In skeletal muscle, Dnmt1 mRNA expression (Figure 5B) was lower in the obesogenic diet group; notably, EC treatment blocked this decrease, and expression levels did not differ from those of the control group. The same pattern was observed for *Dnmt3a* (Figure 5D) and *Dnmt3b* (Figure 5F), where EC treatment prevented the obesogenic diet–induced reduction in mRNA expression.

### 3.6. Peroxisome Proliferator-Activated Receptor Gamma Coactivator 1 Alpha (Pgc-1α) Expression

**Figure 6:**
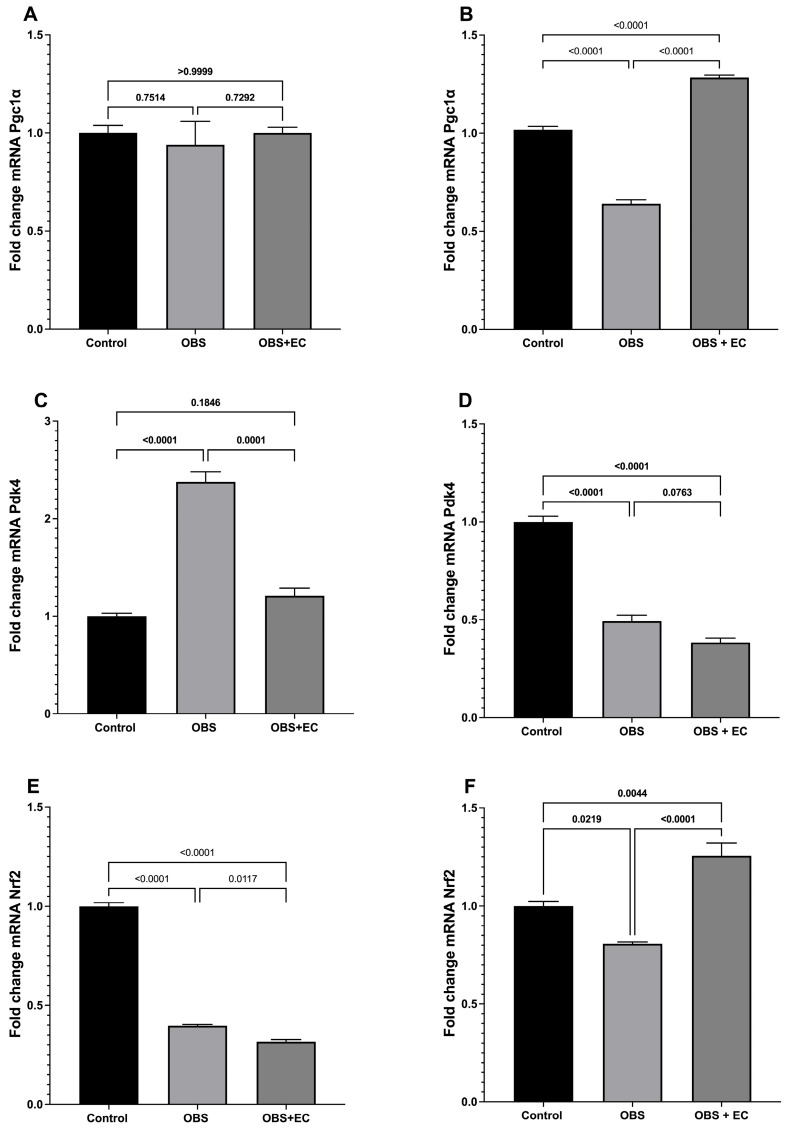
Analysis of Peroxisome proliferator-activated receptor gamma coactivator 1 alpha (*Pgc-1α*), Pyruvate dehydrogenase kinase isozyme 4 (*Pdk4*), and nuclear factor erythroid 2-related factor 2 (*Nrf2*) expression in visceral fat (**A**,**C**,**E**) and skeletal muscle (**B**,**D**,**F**) (*n* = 4 for control group, *n* = 6 for OBS, and *n* = 6 for OBS + EC). Data are represented as means ± SEM.

Pgc-1α is a key regulator of mitochondrial biogenesis and energy metabolism [24]. Our results showed that *Pgc-1α* expression in visceral fat did not change in the obesogenic context (Figure 6A); however, in skeletal muscle (Figure 6B), expression was significantly lower in the obese group. Interestingly, treatment with EC prevents this decrease; *Pgc-1a* expression increases significantly, to a greater extent than in the control group.

### 3.7. Pyruvate Dehydrogenase Kinase Isozyme 4 (Pdk4) Expression

Pdk4 plays an important role in metabolic flexibility by regulating pyruvate metabolism and epigenetically linked energy homeostasis [25]. We observed a significant increase in *Pdk4* in visceral fat in the obesogenic group. This increase was significantly blocked in the obesogenic diets plus EC group, with no difference relative to the control group (Figure 6C). On the other hand, the expression is lower in the skeletal muscle of the obesogenic diet group than in the control group (Figure 6D); however, EC treatment did not reverse this decrease.

### 3.8. Nuclear Factor Erythroid 2–Related Factor 2 (Nrf2) Expression

Nrf2 is a transcription factor involved in antioxidant defense and epigenetic regulation of cellular stress responses [26]. We observed a decrease in *Nrf2* expression in VAT in the obese group; the EC group showed a greater decrease (Figure 6E). Meanwhile, in skeletal muscle, *Nrf2* expression was significantly reduced in the obese group (Figure 6F). EC prevents this reduction and maintains *Nrf2* expression at levels higher than those in the control group.

### 3.9. Mitochondrial Number Changes (Relative mtDNA Content)

We evaluate the ratio between mitochondrial DNA encoding 16S ribosomal RNA (*mt-Rnr2*) (16S rRNA) and nuclear DNA encoding hexokinase 2 (*HK2*) to estimate relative mitochondrial DNA content (mtDNA/nDNA ratio) [27]. Analyses were performed using control: *n* = 4, obesogenic diet: *n* = 6, obesogenic diet + EC: *n* = 6, with three technical replicates per sample.

**Figure 7:**
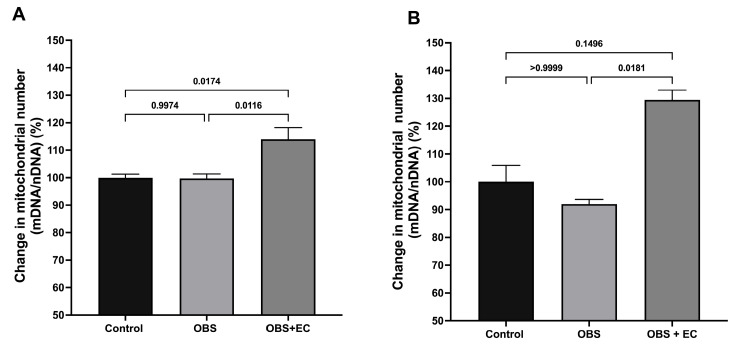
Analysis of mitochondrial DNA vs. nuclear DNA (mtDNA/nDNA ratio) and induced changes by ingestion of HDF and the EC-induced changes in (**A**) visceral fat and (**B**) skeletal muscle. (*n* = 4 for control group, *n* = 6 for OBS, and *n* = 6 for OBS + EC). Data are represented as means ± SEM.

Figure 7A shows the percentage change in the mtDNA/nDNA ratio in VAT. Obesogenic HFD does not alter relative mtDNA content; however, EC treatment causes a significant increase of approximately 15%. Interestingly, in skeletal muscle, although the obesogenic diet induced a slight but not substantial decrease in relative mtDNA, EC treatment caused a significant increase of nearly 25% in mtDNA content in this tissue (Figure 7B).

### 3.10. Leptin Expression Changes

**Figure 8:**
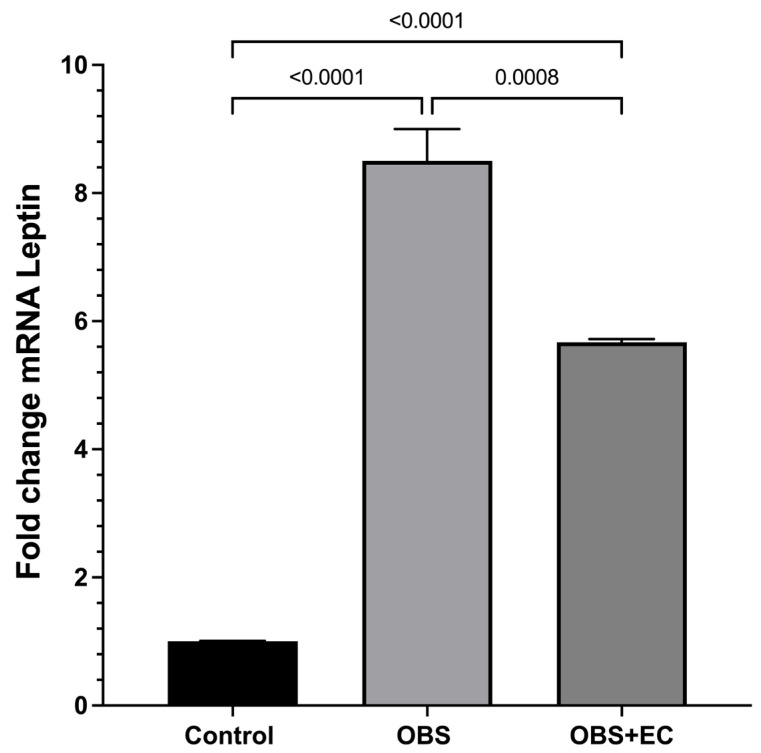
Analysis of leptin gene expression and induced changes by ingestion of HDF and the EC-induced changes. (*n* = 4 for control group, *n* = 6 for OBS and *n* = 6 for OBS + EC). Data are represented as means ± SEM.

Leptin is a key adipokine that regulates energy homeostasis and obesity-associated metabolic alterations [19]. To investigate epigenetic secondary changes induced by HFD and the EC effects on this phenomenon, we examined *leptin* mRNA expression following HFD ingestion. The results showed a remarkable and significant increase in VAT leptin expression, which was significantly reduced by EC treatment, suggesting a positive modulatory effect of the flavanol (Figure 8).

## 4. Discussion

The in silico results reported here showed that within DNMT enzymes, two main sites are essential to allow the methylation reaction: First, the site for the cofactor binding, i.e., S-adenosyl methionine (SAM), and second, the catalytic site, known as the SPC site. Analysis of molecular dynamics simulations showed that EC binds to the three evaluated DNMT isoforms via hydrogen-bond formation throughout the simulations. The main difference in binding among EC isoforms was the site at which this molecule formed favorable interactions. In this sense, EC induced hydrogen bonding throughout the dynamics, with three crucial residues of the DNMT1 cofactor-binding site. Based on these results, the flavanol EC may compete with SAM for the cofactor-binding site in this isoform.

Regarding DNMT3a, EC most often adopts a binding conformation near both sites susceptible to inhibition, as evidenced by hydrogen-bonding analysis. This may be relevant to the modulation of the enzyme’s function; EC interaction with Cys706 in the catalytic site could block the nucleophilic attack by this residue on the target cytosine [20].

Finally, EC occupies the cofactor-binding site of DNMT3b, adopting a conformation that enables hydrogen-bond formation with residues at this site in approximately 60% of the time, suggesting the potential to act as a blocker at this site.

Altogether, the in silico analysis of the interaction of EC and DNMTs strongly suggests that the flavanol could block the enzymes’ activities. This assumption was corroborated by the in vitro study, which showed a significant, concentration-dependent decrease in enzyme activity induced by EC.

On the other hand, in the mouse model, EC administration reduced body weight in obese mice. Previous studies have shown that EC promotes adipose tissue browning by increasing the expression of key genes related to thermogenesis, promoting the phosphorylation of regulators of fatty acid oxidation, and decreasing triglyceride levels. These effects contribute to body weight reduction by increasing energy expenditure and lipid mobilization [9].

In muscle, after EC treatment, an increased expression of *Pgc-1α* and *Nrf2* was observed in obese animals, along with increased mitochondrial copy number and decreased Pdk4 expression. These findings are consistent with previous studies reporting that DNA methylation regulates genes associated with mitochondrial biogenesis and energy homeostasis [21,22]. Our results showed that, after EC treatment, increased mitochondrial DNA copy number (relative mtDNA content (mtDNA/nDNA ratio) in VAT and skeletal muscle, and decreased leptin expression were observed in visceral adipose tissue. In mouse models, a high-fat diet has been shown to increase leptin expression via promoter demethylation [23]. The decrease in leptin expression may be associated with epigenetic changes that affect adipose tissue function in the context of obesity and energy metabolism. However, the effect observed in this tissue was smaller than that observed in muscle, suggesting differences in epigenetic susceptibility between the two tissues. The results agree with those from Milenkovic et al., suggesting that EC is an epigenetic modulator [28,29].

To determine the possible role of EC in the epigenetic regulation of energy metabolism, we analyzed global DNA methylation in muscle and visceral adipose tissue. Global methylation reflects the total methylation level of a cell and provides an overview of potential epigenetic changes. However, epigenetic regulation is a dynamic process in which specific genes can be methylated while others can be demethylated to modulate gene expression [5,30].

Our results showed that obesity induced in the mouse model reduced global DNA methylation in both tissues. Interestingly, EC treatment restored global methylation levels in muscle to levels comparable to the control group, suggesting that EC may modulate energy metabolism through epigenetic mechanisms.

In contrast, global DNA methylation in VAT showed no significant differences between the EC-treated groups and the obese group, which was unexpected. Previous studies have shown that diets rich in fatty acids, such as palmitic acid, can induce hypermethylation in adipose tissue in mouse models, whereas our model did not show this [31]. In mouse models subjected to caloric restriction, differential responses across tissues have been observed, with some tissues showing hypermethylation and others hypomethylation [32]. Although a trend toward reduced global methylation was observed in the obese group treated with EC in our study, this decrease was not statistically significant. These results suggest that the impact of EC on global methylation in visceral adipose tissue may depend on additional factors.

The existing body of research on global methylation has yielded inconclusive results, underscoring the necessity for a targeted approach to address epigenetic modifications. Our data suggest that EC’s effects on epigenetic regulation are tissue-specific and may involve distinct molecular mechanisms. It is plausible that EC modulates methylation selectively at specific genes. For instance, saturated fatty acids and elaidic acid have been observed to induce hypomethylation of tumor necrosis factor (*Tnf*) and hypermethylation of Peroxisome Proliferator-activated receptor gamma 1 (*Pparg1*), thereby altering their gene expression and promoting inflammation in adipose tissue [33]. This finding underscores that the epigenetic response to EC may be contingent on gene specificity and the tissue’s metabolic context.

Given the established correlations between global DNA hypomethylation, genomic instability, and disease development, including cancer [34,35], it is imperative to elucidate the implications of these changes for long-term metabolic homeostasis. The results of this study indicate a correlation between *Dnmts* expression and global DNA methylation levels. However, these results do not necessarily imply a direct correlation with localized hypermethylation in specific genome regions.

This study also analyzed *Dnmt* gene expression, specifically *Dnmt1*, in VAT [36]. A significant difference in this enzyme’s expression was observed between the control and obese groups, consistent with previous studies reporting lower DNMT1 expression in obese individuals than in healthy individuals [37]. However, no increase in this gene’s expression was observed after EC treatment, suggesting that the flavanol may act through other mechanisms, possibly by inhibiting DNMT1 activity rather than affecting its expression, as reported for similar compounds such as epigallocatechin [38].

The available evidence for DNMT3a is conflicting. However, DNMTs play distinct roles during adipogenic differentiation, depending on cell type, complicating the overall understanding of these molecules. Our data show a decrease in *Dnmt3a* expression in the obese group, which contrasts with other studies reporting its overexpression in obese models, an association linked to the development of insulin resistance in visceral adipose tissue. In contrast, previous research has shown that DNMT3a deficiency predisposes to obesity, even under normal dietary conditions. This mechanism is explained by the fact that loss of DNMT3a leads to a significant increase in the stem cell pool and inflammatory genotype in adipose tissue [39].

Interestingly, EC treatment further reduced *Dnmt3a* expression, suggesting a protective effect against diet-induced responses and impaired glucose tolerance. However, restoration of expression to levels comparable to the control group was not observed.

Dnmt3b expression also decreased in the visceral adipose tissue of the obese model, thereby precluding detection of significant differences between the experimental groups. This gene is predominantly expressed in white adipose tissue, which complicates the evaluation of EC effects in this context [40].

These results may be related to the diet used in the experimental models. Previous studies have shown that prolonged high-fat, high-calorie diets (at least 36 weeks) are necessary to observe significant effects on adipose tissue [41]. Despite the absence of a clear trend in *Dnmt* gene expression or in global DNA methylation in this study, genes related to mitochondrial metabolism and biogenesis were evaluated.

Adipose tissue plays a central role in energy metabolism and, as a reservoir of triglycerides, functions as an endocrine organ that regulates multiple metabolic processes. Genes such as *Pgc-1α, Pdk4*, and *leptin* are critical for energy homeostasis, thermogenesis, and regulation of glucose and lipid metabolism. Regarding mitochondrial biogenesis-related endpoints, an increase in relative mtDNA content (mtDNA copy number proxy) was observed in the EC-treated group, although *Pgc-1α* expression did not differ significantly between groups. The literature suggests that HFD may reduce the expression of genes involved in oxidative phosphorylation and β-oxidation, thereby impairing glucose tolerance and promoting insulin resistance, mechanisms in which the *Pgc-1α* gene plays a key role [24].

Regarding *Pdk4* expression, an increase was observed in VAT of the obese group, indicating increased metabolic activity in adipose tissue due to the hypercaloric diet [25,42], skeletal muscle showed a decrease in *Pdk4* expression; we do not have a clear explanation for these tissue-dependent differences. The results in skeletal muscle agree with results found in humans, where a high-fat diet induced differential gene expression in skeletal muscle between lean and obese individuals. Obese individuals show reduced or impaired adaptation of oxidative genes (PDK4 and PGC1α). Obese and insulin-resistant individuals fail to adequately regulate these pathways, resulting in metabolic inflexibility and a possible accumulation of lipid intermediates. Interestingly, *Pdk4* expression decreased in the EC-treated group and approached levels comparable to those in the control group. EC treatment does not alter *Pdk4* expression in skeletal muscle [43].

As for leptin, an indicator of body fat, a decrease was observed in the EC-treated group, which could contribute to improved insulin sensitivity, as reported in previous studies [26].

For the *Nrf2* gene, increased expression was observed in the obese group, suggesting adaptation to diet and increased oxidative stress and inflammation in adipose tissue. This finding is consistent with previous studies reporting increased *Nrf2* in obese models [44]. EC reduced the expression of this gene in the treated group, which may suggest a protective effect, although this could not be fully elucidated due to differences in the dietary models used [45].

The results in muscle contrast significantly with those obtained in visceral adipose tissue, where differences between the obese and EC-treated groups were more pronounced. Regarding global DNA methylation, both methylation percentage and *Dnmt* gene expression were lower in the obese group. On the other hand, methylation increased in the EC-treated group, approaching levels observed in the control group, suggesting that EC may positively influence epigenetic regulation in this tissue. This finding is consistent with that reported by Hatazawa et al. [46], who noted that in muscle tissue, decreased global methylation is associated with reduced muscle mass and impaired satellite cell differentiation, a phenomenon observed in the obese group that could be improved by EC treatment.

Regarding the *Dnmt3a* gene, a study by Small et al. [47] indicates that this gene does not affect the energetic state of muscle, supporting the conclusion that EC effects on muscle do not depend on this gene. Furthermore, when analyzing mitochondrial biogenesis -related endpoints and energy metabolism in muscle, a relative mtDNA content (mtDNA copy number proxy) was observed in obesity, which increased in the EC-treated group, even exceeding control levels. This is consistent with the study by Yang et al. [48], who reported reduced Pgc-1α expression in muscle tissue under conditions of obesity and diabetes, supporting the notion that EC may protect mitochondrial biogenesis-related processes.

Analysis of genes involved in energy metabolism, such as *Pdk4* and *Nrf2*, also yielded interesting insights. *Pdk4* expression was decreased in the obese group, suggesting altered glucose oxidation and metabolism, as this gene inhibits pyruvate formation. However, in the EC-treated group, *Pdk4* expression was normalized, suggesting that EC regulates gluconeogenic metabolism. On the other hand, *Nrf2*, a key gene in tissue protection, showed increased expression in the EC-treated group, indicating improved muscle activity and energy metabolism, consistent with previous studies suggesting that Nrf2 overexpression is associated with improved muscle activity [49]. However, the study has limitations, primarily related to the duration of the obesogenic diet in the mouse model, which did not reach the durations used in other studies, in which more pronounced effects on DNA methylation and Dnmt expression are observed. Furthermore, it would be valuable to extend the study model by incorporating restrictive diets and combinations with EC to evaluate synergistic effects that may be potentiated by caloric restriction.

## 5. Conclusions

The main results found in this work are as follows: (1) In silico analyses suggested potential binding of epicatechin to DNMTs (proteins). The computational results are consistent with the possibility that EC may act as a competitive antagonist; the in vitro nuclear-extract assay showed significant, concentration-dependent inhibition of DNMT activity. (2) An obesogenic diet decreased the percentage of methylated DNA, and the EC-induced effects are tissue-specific (different between visceral fat and skeletal muscle). (3) The obesogenic process decreased the mRNA expression of *Dnmt1*, *Dnmt3a*, and *Dnmt3b*. The effects of the flavanol EC are also tissue-specific; only in skeletal muscle were they opposite to those induced by the obesogenic diet. (4) Changes in the expression of *Pgc-1a, Nrf2*, and *Pkd4* also differ in the two analyzed tissues, suggesting a particular modulation of effects. (5) Relative mtDNA content (mtDNA/nDNA ratio) was not altered by the obesogenic diet; however, EC increased mtDNA/nDNA ratio in both visceral fat and skeletal muscle, suggesting a positive modulation of EC, probably increasing metabolic rates.

Despite discrepancies in some genes, this study elucidates the role of EC as a potential treatment for obesity. It is suggested that more in-depth investigations be conducted, expanding the number of genes studied, evaluating their promoters, and analyzing protein levels and functions, including proteomic analyses. This could confirm EC’s protective effects against obesity and elucidate its role in metabolic regulation. However, it is clear that epicatechin warrants further study as a potential epigenetic modulator.

## Figures and Tables

**Table 1:** Oligonucleotides used.

Enzyme	Oligonucleotide 5’→3’
*Dnmt1-F*	CCTAGTTCCGTGGCTACGAGGAGAA
*Dnmt1-R*	TCTCTCTCCTCTGCAGCCGACTCA
*Dnmt3A-F*	GCCGAATTGTGTCTTGGTGGATGACA
*Dnmt3A-R*	CCTGGTGGAATGCACTGCAGAAGGA
*Dnmt3B-F*	TTCAGTGACCAGTCCTCAGACACGAA
*Dnmt3B-R*	TCAGAAGGCTGGAGACCTCCCTCTT
*Hk2-F*	GCCAGCCTCTCCTGATTTTAGTGT
*Hk2-R*	GGGAACACAAAAGACCTCTTCTGG
*Pgc-1a-F*	ACCCACAGGATCAGAACAAACCCT
*Pgc-1a-R*	TTGGTGTGAGGAGGGTCATC
*Pdk4-F*	GTCGAGCATCAAGAAAACC
*Pdk4-R*	GCGGTCAGTAATCCTCAGAG
*Lep-F*	TGGCTTTGGTCCTATCTGTC
*Lep-R*	TCCTGGTGACAATGGTCTTG
*Actb-F*	GGCTGTATTCCCCTCCATCG
*Actb-R*	CCAGTTGGTAACAATGCCATGT

## Data Availability

Data is contained within the article.
